# Olfaction preservation in olfactory groove meningiomas: a systematic review

**DOI:** 10.1007/s10143-023-02096-z

**Published:** 2023-07-27

**Authors:** Nicholas G. Candy, Dominik Hinder, Alistair K. Jukes, Peter-John Wormald, Alkis J. Psaltis

**Affiliations:** 1 Department of Surgery Otolaryngology, Head and Neck Surgery, The University of Adelaide, Basil Hetzel Institute for Translational Research, Woodville South, Adelaide, Australia; 2 Department of Surgery Otorhinolaryngology Head and Neck Surgery, Queen Elizabeth Hospital, Adelaide, SA Australia; 3 Department of Neurosurgery, Royal Adelaide Hospital, Adelaide, Australia

**Keywords:** Endoscopic surgery, Meningioma, Olfaction, Neurosurgery

## Abstract

Olfactory groove meningiomas (OGM) are a skull base neoplasm that represents between 8 and 13% of all intracranial meningiomas. Approach selection focuses on achieving frontal lobe decompression, gross total resection and vision preservation. Recently, there has been a focus on olfaction and considering its preservation as a quality-of-life outcome measure. An electronic search of the databases Medline, Scopus, Embase, Web of Science and Cochrane library databases was performed and data extracted according 2020 Preferred Reporting Items of Systematic Reviews and Meta-Analyses (PRISMA) statement. Six articles were selected for inclusion mainly based due to reporting quantitative outcomes for olfaction assessed by a smell identification test (e.g. sniffin’ sticks). Objective olfaction preservation can be achieved with a variety of surgical approaches. More research which includes objective assessment of olfactory function and ideally as well QoL outcome measures is needed to further optimize the treatment pathways in OGM patients.

## Introduction

Olfactory groove meningiomas (OGM) are a skull base neoplasm that arises from the lamina cribrosa and frontoethmoidal suture. They represent between 8 and 13% of all intracranial meningiomas [1]. Patients can present with headache, personality and cognitive changes, visual impairment and alterations in their sense of smell [2]. When symptomatic, management of these tumours is primarily surgical. Broadly, these tumours can be approached endonasally with an endoscopic endonasal approach (EEA), or transcranially through a craniotomy [3]. Traditionally, the approach selection has focused on achieving the surgical goals of frontal lobe decompression, gross total resection, vision preservation and enhanced recovery. In recent years, there has been an increase in a number of articles examining olfaction and its preservation as a quality-of-life outcome measure [4].

The goal of this article is to systematically review the available literature examining olfaction outcomes in patients with olfactory groove meningiomas and recommend a strategy to approach these tumours.

## Methods

### Literature search

A search strategy was devised according to the 2020 Preferred Reporting Items of Systematic Reviews and Meta-Analyses (PRISMA) statement. An electronic search of the databases Medline, Scopus, Embase, Web of Science and Cochrane library databases was performed from January 1996 until 26th of May 2023. To identify articles investigating olfactory outcomes in olfactory groove meningioma resection, the following search terms were applied: (((olfaction OR smell) AND (olfactory groove OR anterior skull base)) AND (meningioma)) AND (outcome) with prior checking in the MeSH database to include synonyms.

The database search was further supplemented by a search of the reference lists of included studies as well as checking the related article function provided by each database. Titles and abstracts were screened to identify potentially relevant studies. All potentially relevant articles, or articles where it was unclear based on the abstract, were assessed by reviews of the full-text articles.

Articles were eligible if they (1) included only anterior skull base meningiomas, (2) reported outcomes for olfaction assessed by a smell identification test (e.g. sniffin’ sticks), (3) presented original data on patients and (4) included patients who had surgery as primary management. Studies were excluded when (1) results did not specifically detail the surgical outcomes for olfactory groove meningioma resection and (2) did not demonstrate systematic assessment of olfaction pre-operatively and post-operatively.

### Data extraction

All data was reviewed independently by 2 authors (NC and DH), and discrepancies were cross-checked in a consensus meeting.

The following data was obtained from the included studies: mean age, gender, number of patients, tumour size, peritumoural oedema, pre-operative olfaction assessment, visual assessment, type of approach, degree of resection, post-operative olfaction in patients with normal olfaction pre-operatively and visual outcome in patients with abnormal vision pre-operatively.

### Quality assessment

We used a modified quality assessment tool incorporating the Cochrane Collaboration tool to assess the methodological quality of the included articles [5]. The quality assessment tool assessed the following: demographic details, pre-operative variables, post-operative variables and follow-up (refer to Table 1). The same two authors (NC and DH) then evaluated the risk of bias in the individual articles using a modified version of the Cochrane Collaboration method (refer to Table 2). Discrepancies were resolved between two authors NC and DH.

**Table 1:** Quality assessment tool

*Quality category*	*Questions*	*Response*		
		Yes	No	Unclear
Demographic details	Is the age and gender for each surgical group defined?			
	Is the date range of the surgical series defined?			
	Are the number of patients examined clearly defined?			
	Is it defined if these cases are sequential or part of a larger surgical series?			
Pre-operative variables	Is pre-operative olfaction assessment defined clearly?			
	Is olfaction assessed with an objective quantitative scale?			
	Is vision assessed quantitatively?			
	Is tumour size and location defined clearly for patients with OGMs?			
	Is presence of peritumoural oedema defined for all patients with OGM?			
Post-operative variables	Is the degree of resection defined clearly for all patients?			
	Is the type of approach defined for all patients?			
	Is the relative change in olfaction clearly defined for all patients?			
	Are the visual outcomes clearly defined?			
Follow-up	Is olfaction re-examined after a period of long-term follow-up?

**Table 2:** Grading of quality assessment

*Quality category*	Poor	Moderate	Good
Demographic details	< 4 criteria	3 of 4 criteria	4 of 4 criteria
Pre-operative variables	< 5 criteria	4 of 5 criteria	5 of 5 criteria
Post-operative variables	< 4 criteria	3 of 4 criteria	4 of 4 criteria
Follow-up	0 criteria		1 of 1 criteria

## Results

### Study selection

**Fig. 1:**
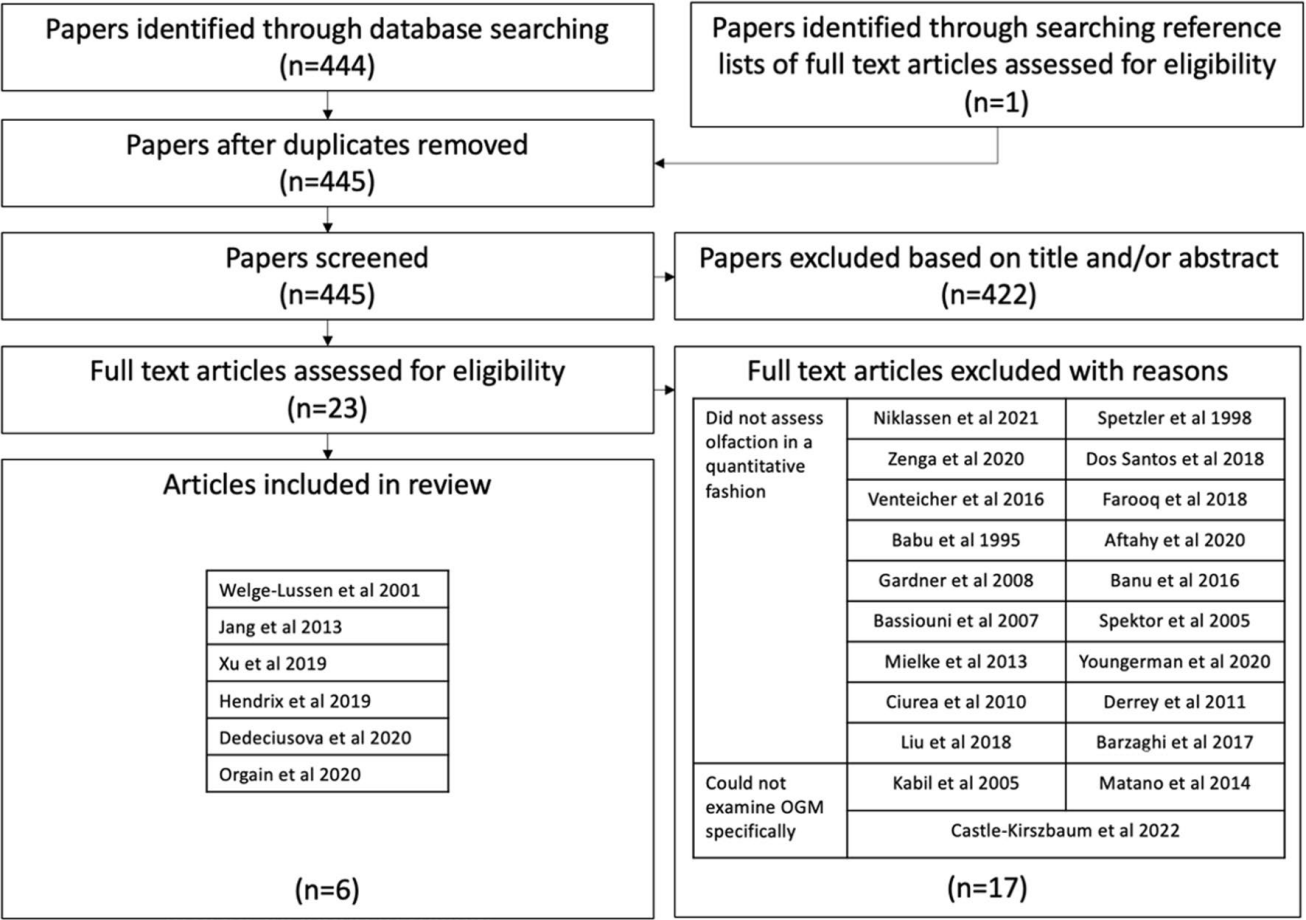
PRISMA flow diagram demonstrating study selection process

From the literature search, 444 articles were identified through searching Medline, Scopus, Embase, Web of Science and Cochrane library databases (refer to Fig. 1). One article was identified through searching reference lists of full-text articles assessed for eligibility. After duplicates had been removed, 445 articles were screened with 422 being excluded based on the content of the title or the abstract. The most common reason for exclusion was absent assessment of olfactory function. Twenty-three articles were read in full with 6 articles [4, 6–10] being selected for inclusion. Seventeen articles [1, 3, 11–29] were excluded. Of these, 14 [1, 3, 11–14, 16–19, 21, 22, 24–29] did not assess olfaction in a quantitative or systematic fashion, and 3 articles [15, 20, 23] presented data on a cohort that did not enable specific examination of patients with OGMs.

### Study characteristics

Welge-Luessen et al. [9] reported on 12 patients with OGMs operated on between 1998 and 2000 at 5 different hospitals in Switzerland, Germany and Austria. Surgery was performed by either a frontal or bifrontal approach. Patients were selected from a larger cohort if they were willing to participate in the study. Olfactory function was assessed using the sniffin’ stick test [30] including lateralized testing and scored out of a total of 48 points, with scores below 16 defined as functional anosmia and scores below 31 as hyposmia. Post-operative olfaction was examined between 2 and 12 weeks after surgery.

Jang et al. [7] reported on 40 patients with OGMs operated on between January 1994 and March 2012 at the Department of Neurosurgery, Chonnam National University Hwasun Hospital, Gwangju, South Korea. Tumour resection was done by a bifrontal or frontolateral approach. Patients were recruited sequentially and formed a part of a larger series of intracranial meningiomas operated on during the same time period. Olfactory function was examined using the Korean version of the sniffin’ stick test [31] and scored out of a total 48 points. Anosmia was defined as scores less then 15 on both sides. A score over 16 on at least one side was defined as hyposmia or normal. Post-operative olfaction was assessed within 2 weeks after surgery.

Xu et al. [10] reported on 54 patients with anterior skull base meningiomas operated on between July 2013 and June 2017 at the Department of Neurosurgery of Huashan Hospital, Shanghai, China. All patients were operated on by using a modified bifrontal approach. Patients were clearly divided into OGMs (29 patients), planum sphenoidale meningiomas and tuberculum sellae meningiomas. Patients with tumours on one side, or recurrent cases, were excluded. Olfaction was tested with 5 common odours in a blinded fashion, with the patient being asked to subjectively report their olfactory outcome as anosmic, hyposmic or normal. Post-operative follow–up was assessed at follow-up at least up to 12 months.

Hendrix et al. [6] reported on 23 patients with olfactory system affecting group meningiomas, of which 9 patients had OGMs. Patients were operated on between January 2015 and January 2016 at the Department of Neurosurgery, Saarland University Medical Centre, Saarland, Germany. Surgery was performed by using an endoscopic-assisted supraorbital frontolateral approach. Patients were not clearly divided between OGMs and other meningiomas and were excluded if they had pre-existing bilateral anosmia from an unrelated pathology. Olfaction was assessed using the sniffin’ stick test and scored as anosmic, hyposmic or normosic (0–6, 7–10 and 11–12, respectively). The 9 patients with OGMs were further divided into 15 olfactory tracts based on whether they were involved by unilateral or bilateral tumour. Post-operative outcomes are then reported per olfactory tract as oppose to per patient. Post-operative olfaction was assessed post-operatively and during follow-up, but this interval is not defined.

Orgain et al. [8] reported on 4 patients with OGMs operated on during an undefined time period in Sydney, Australia or Pennsylvania, USA. Patients were included if they underwent surgery through a unilateral EEA and had a unilateral tumour. The contralateral side of the nose and sinuses stayed untouched during the procedure. In 2 patients, olfaction was assessed pre-operatively by using a six-point olfactory symptom score. The other 2 patients had no standardized pre-operative olfaction assessment. In all 4 patients, olfaction was tested post-operatively by using the sniffin’ stick 12. Therefore, change in olfaction is unexaminable for 2 patients due to the absence of pre-operative testing with sniffin’ sticks.

Dedeciusova et al. [4] reported on 13 patients with OGMs who underwent surgery between December 2013 and December 2017 at the Charles University and Military Hospital, Prague, Czech Republic. All patients were operated by using a unilateral craniotomy. Patients with known anosmia secondary to unrelated pathology were excluded. Olfaction was assessed using sniffin’ stick test and scored according to age- and sex-based percentiles. Post-operative olfaction was examined within 7 days of surgery at 12 months after surgery.

### Pre-operative findings

Pre-operative findings are reported variably between the included articles and are displayed in Table 3.

**Table 3:** Table of available demographic data from the included articles

	Mean age (years)	Gender	Number of patients	Tumour size	Peritumoural oedema	Pre-operative olfaction assessment	Pre-operative olfaction score	Visual function
Welge-Luessen et al. 2001	51	7/12 F (58%)	12	2–5 cm	N/A	Sniffin’ stick test	4/12 (33%) normal bilaterally 2/12 (17%) hyposmia bilaterally 4/12 (33%) anosmia bilaterally 1/12 (8%) anosmia ipsilateral, normal contralateral 1/12 (8%) anosmia ipsilateral, hyposmia contralateral	N/A
Jang et al. 2013	53	17/40 F (42.5%)	40	4.59 cm^3	28/40 (70%)	Korean version of sniffin’ Stick test	17/40 (67.5%) anosmia/hyposmia 23/40 (32.5%) normal	21/40 (52.5%) visual impairment
Xu et al. 2019	54.9	18/29 F (62%)	29	4.31 cm^3	23/29 (79.3%)	Blinded testing of 5 odours with subjective olfactory outcome	17/29 (58.6%) anosmia/hyposmia 12/29 (41.3%) normal	6/29 (20.7%)
Hendrix et al. 2019	61.5	N/A	9^$^ 15^*^	N/A	N/A	Sniffin’ stick test	6/15* (40%) anosmia 5/15* (33%) hyposmia 4/15* (27%) normal	N/A
Orgain et al. 2020	41.5	1/4 F (25%)	4	6.5 cm^3	N/A	Sniffin’ stick test + 6 point olfactory symptom score	4 patients normal (only 2 assessed with sniffin’ stick test)	N/A
Dedeciusova et al. 2020	59	10/13 F (76%)	13	10.9 cm^3	9/13 (69%)	Sniffin’ stick test	3/13 (23%) anosmia 2/13 (15%) impaired 8/13 (61%) normal	2/13 impaired (15.4%)

Absolute number reported if available, and then percentage of cohort

Abbreviation: *F*, female; *N/A*, not assessable

^*^Reported as absolute number of olfactory tracts affected by olfactory groove meningioma. ^$^Defined olfactory groove meningioma as tumours also extending to the planum and tuberculum sellae

### Olfactory assessment

The method of olfactory assessment is reported variably in all 6 articles [4, 6–10]. Five of the included articles [4, 6–9] examine olfaction using the sniffin’ stick test. Of these articles, 1 article [7] uses a Korean version of the original as reported by Hummel et al. [30] in 2001. One of the articles [6] uses a unique scoring scheme to what was outlined in Hummel et al. The remaining article [10] systematically assesses olfaction but in a subjective fashion by using 5 different odours and asking the patient to grade their olfaction.

### Post-operative findings

Post-operative findings are reported variably between the included articles and are displayed in Table 4.

**Table 4:** Table of available post-operative outcome data

	Degree of resection	Type of approach	Post-operative olfaction in patients with normal olfaction pre-operatively	Visual outcomes in patients with abnormal vision
Welge-Luessen et al. 2001	12/12 (100%)	12/12 Bifrontal or unifrontal	6/6 (100%) new anosmia ipsilateral to tumour 1/8 (12.5%) new hyposmia contralateral to tumour 4/8 (50%) new anosmia contralateral to tumour	N/A
Jang et al. 2013	Simpson I and II resection 37/40 (92.5%)	21/40 (52.5%) frontolateral 19/40 (47.5%) bifrontal	22/23 (95%) remained normal	2/40 (5%) deteriorated Unknown if patients improved
Xu et al. 2019	Simpson I and II resection 29/29 (100%)	29/29 (100%) bifrontal	6/12 (50%) remained normal 2/12 (16%) new hyposmia	5/6 (83%) improved 1/6 (17%) deteriorated
Hendrix et al. 2019	N/A	9/9 (100%) endoscopic-assisted supraorbital craniotomy	2/15 normosmic and 2/15 hyposmic nerves became anosmic	N/A
Orgain et al. 2020	4/4 GTR	4/4 (100%) EEA	SS-12 $9+/-1.4$ 75% subjectively described a mild impairment or better	N/A
Dedeciusova et al. 2020	13/13 (100%) GTR	13/13 (100%) frontolateral	5/8 (62.5%) remained normal 1/8 (12.5%) new hyposmia 2/8 (25%) new anosmia 1/2 (50%) remained hyposmic 1/2 (50%) new anosmia 1/3 (33%) improved to hyposmia 2/3 (66%) remained anosmic	1/13 (7.6%) new visual deterioration N/A for other cases

Data reported as either absolute number of patient within total cohort and associated percentage, or as mean +/- standard deviation

Abbreviation: *N/A*, not available; *EEA*, endoscopic endonasal approach; *GTR*, gross total resection

### Rates of olfaction preservation

Rates of olfaction preservation were reported differently in all 6 included articles [4, 6–10]. Welge-Luessen et al. [9] reported olfaction outcome for each nostril in reference to the tumour location, with 100% of patients with normal olfaction pre-operatively ipsilateral to the tumour developing anosmia post-operatively. 62.5% of the patients with preserved olfaction contralateral to the tumour suffered a deterioration either hyposmia (12.5%) or anosmia (50%) post-operatively. Jang et al. [7] reported olfaction as a summary measure documenting 95% of patients remained with normal olfaction. Xu et al. [10] reported in a similar method with 50% of patients with normal olfaction pre-operatively maintaining this post-operatively. Hendrix et al. [6] reported olfaction in a confusing method by olfactory tract, but not defining the laterality. Orgain et al. [8] reported the mean score from sniffin’ stick 12, but because 50% of the patients did not have sniffin’ stick test pre-operatively, the relative change could not be calculated. Dedeciusova et al. [4] reported the summary outcome for all patients pre-operatively and post-operatively, with 62.5% of patients with normal olfaction pre-operatively remaining normal post-operatively. One patient with anosmia pre-operatively improved to hyposmia post-operatively.

### Study quality

Overall study quality was determined to be poor in 4 articles [6–9] and good in 2 articles [4, 10] (refer to Table 5). Common features between articles of low study quality included the following: quantitatively examining vision pre-operatively, not defining the relative olfaction change between patients clearly and defining the visual outcome clearly.

**Table 5:** Quality assessment consensus table

Paper	Demographic details	Pre-operative variables	Post-operative variables	Follow-up	Overall quality
Welge-Luessen et al. 2001	Good	Poor	Poor	Poor	Poor
Jang et al. 2013	Good	Good	Poor	Poor	Poor
Xu et al. 2019	Good	Moderate	Good	Good	Good
Hendrix et al. 2019	Moderate	Poor	Poor	Good	Poor
Orgain et al. 2020	Moderate	Poor	Poor	Good	Poor
Dedeciusova et al. 2020	Good	Good	Moderate	Good	Good

## Discussion

Rates of olfaction preservation were reported in all 6 articles [4, 6–10] with varying success. Rates of preserved olfaction ranged between 95^7^ and 50%^10^ in the articles were it could be clearly interpreted. The literature does show that olfaction preservation is possible in carefully selected patients.

Although olfaction preservation is possible through an EEA as demonstrated by one of the articles [8], this would have very narrow selection criteria compared to an open approach. The authors looked at 4 patients with a unilateral EEA leaving the other side of the nose and sinuses completely untouched. Based on the articles, there does not appear to be any major difference with open approach selection with 2 articles [9, 10] being bifrontal or unifrontal, 1 article [6] being an endoscopic assisted supraorbital, 1 article [4] being frontolateral and 1 article [7] being a mixture of bifrontal and frontolateral.

It is important to note that subjective olfaction and objective olfactory testing demonstrate significantly different results. One article [7] found only 15% of patients noted difference in olfaction but found 67.5% of patients had objective olfaction disturbance on quantitative testing. This points to the importance of objectively assessing patients with OGMs pre-operatively as patient reported olfaction appears to be unreliable.

The EANS Skull Base Section published a systematic review and meta-analysis on the different microsurgical transcranial approaches and EEAs for management of OGMs in 2022 [32]. They examined olfactory worsening in the included papers but did not differentiate between patients’ subjective olfactory sense and objective quantitative testing. This does question the utility of the meta-analysis performed given the variability between subjective and objective olfaction. Alternatively, it is possible that objective assessment demonstrating olfactory disturbance in the asymptomatic patient is not clinically significant given it does not affect patient quality of life. Furthermore, an important consideration is whether certain approaches deliver better cognitive outcomes. Traditionally, EEA was advocated as a way to reduce frontal lobe retraction and potentially improve cognitive outcomes compared to microsurgical transcranial approaches. However, the review published by the EANS Skull Base Section did not find robust evidence to demonstrate this difference.

Articles that consider olfactory preservation as a primary surgical goal along with complete resection, vision preservation and cognitive outcome are beginning to appear in the literature [3]. Furthermore, there are case reports that demonstrate the possibility of olfaction improvement in patients with pre-operative anosmia who had their olfactory structures preserved during surgery [19, 25]. Despite the increased awareness of olfactory outcomes, there are only a few of these articles have objective testing of the olfactory function included in the pre- and post-operative assessment of the patients.

Although beyond the scope of this article, stereotactic radiosurgery (SRS) as primary management for OGMs is an alternative management to surgical resection in appropriately selected patients. An international, multicentre study [33] reported in 2021 a rate of new olfactory dysfunction of 2%. These patients were examined either objectively or subjectively, making meaningful conclusions difficult. However, it does show that SRS may be an appropriate treatment modality to consider in some patients to facilitate olfaction preservation.

Ultimately, more focused research into olfactory outcomes for the different management modalities needs to be undertaken. Routine objective assessment of patient’s olfaction will then allow a better understanding about how changes to olfaction may affect a patient’s quality of life.

### Limitations

The main limitations of the included articles are related to the variable reporting of outcome variables as well as variation in the way the outcome is reported. This makes direct comparisons between articles difficult.

## Conclusion

We have demonstrated that objective olfaction preservation can be achieved between a variety of surgical approaches. More research which includes objective assessment of olfactory function and ideally as well QoL outcome measures is needed to further optimize the treatment pathways in OGM patients.

## Data Availability

Not applicable.
